# Different regulation of *PARP1, PARP2, PARP3* and *TRPM2* genes expression in acute myeloid leukemia cells

**DOI:** 10.1186/s12885-020-06903-4

**Published:** 2020-05-18

**Authors:** Paulina Gil-Kulik, Ewa Dudzińska, Elżbieta Radzikowska-Büchner, Joanna Wawer, Mariusz Jojczuk, Adam Nogalski, Genowefa Anna Wawer, Marcin Feldo, Wojciech Kocki, Maria Cioch, Anna Bogucka-Kocka, Mansur Rahnama, Janusz Kocki

**Affiliations:** 1 Department of Clinical Genetics, Medical University of Lublin, 11 Radziwillowska Str, 20-080 Lublin, Poland; 2 Department of Public Health, Faculty of Health Sciences, Medical University of Lublin, 1 Chodźki Str, 20-093 Lublin, Poland; 3 Department of Plastic Surgery, Saint Elizabeth’s Hospital, 1 Goszczynskiego Str, 02-616 Warsaw, Poland; 4 Chair and Department of Trauma Surgery and Emergency Medicine, Medical University of Lublin, 16 Staszica Str, 20-081 Lublin, Poland; 5 Department of Foreign Languages Medical University of Lublin, 4 Jaczewskiego Str, 20-090 Lublin, Poland; 6 Chair and Department of Vascular Surgery and Angiology, Medical University of Lublin, 11 Staszica Str, 20-081 Lublin, Poland; 7 Department of Architecture and Urban Planning, University of Technology, 40 Nadbystrzycka Str, 20-618 Lublin, Poland; 8 Chair and Department of Hematooncology and Bone Marrow Transplantation, Medical University of Lublin, 11 Staszica Str, 20-081 Lublin, Poland; 9 Chair and Department of Biology and Genetics, Medical University of Lublin, 4a Chodźki Str, 20-093 Lublin, Poland; 10 Chair and Department of Dental Surgery Medical University of Lublin, 7 Karmelicka Str, 20-081 Lublin, Poland

**Keywords:** *PARP1*, *PARP2*, *PARP3*, *TRPM2* gene expression, AML, Hematopoietic stem cells

## Abstract

**Background:**

Acute myeloid leukemia (AML) is a heterogenic lethal disorder characterized by the accumulation of abnormal myeloid progenitor cells in the bone marrow which results in hematopoietic failure. Despite various efforts in detection and treatment, many patients with AML die of this cancer. That is why it is important to develop novel therapeutic options, employing strategic target genes involved in apoptosis and tumor progression.

**Methods:**

The aim of the study was to evaluate *PARP1, PARP2, PARP3,* and *TRPM2* gene expression at mRNA level using qPCR method in the cells of hematopoietic system of the bone marrow in patients with acute myeloid leukemia, bone marrow collected from healthy patients, peripheral blood of healthy individuals, and hematopoietic stem cells from the peripheral blood after mobilization.

**Results:**

The results found that the bone marrow cells of the patients with acute myeloid leukemia (AML) show overexpression of *PARP1* and *PARP2* genes and decreased *TRPM2* gene expression. In the hematopoietic stem cells derived from the normal marrow and peripheral blood after mobilization, the opposite situation was observed, i.e. *TRPM2* gene showed increased expression while *PARP1* and *PARP2* gene expression was reduced. We observed positive correlations between *PARP1, PARP2, PARP3*, and *TRPM2* genes expression in the group of mature mononuclear cells derived from the peripheral blood and in the group of bone marrow-derived cells. In AML cells significant correlations were not observed between the expression of the examined genes. In addition, we observed that the reduced expression of *TRPM2* and overexpression of *PARP1* are associated with a shorter overall survival of patients, indicating the prognostic significance of these genes expression in AML.

**Conclusions:**

Our research suggests that in physiological conditions in the cells of the hematopoietic system there is mutual positive regulation of *PARP1, PARP2, PARP3,* and *TRPM2* genes expression. *PARP1, PARP2,* and *TRPM2* genes at mRNA level deregulate in acute myeloid leukemia cells.

## Background

Acute myeloid leukemia (AML) is a heterogenic lethal disorder characterized by the accumulation of abnormal myeloid progenitor cells in the bone marrow which results in hematopoietic failure. A major contributing factor to the high mortality rate associated with acute myeloid leukemia is developing resistance to chemotherapy [1]. Despite various efforts in detection and treatment, many patients with AML die of this cancer [1]. Therefore it is important to develop novel therapeutic options, employing strategic target genes involved in apoptosis and tumor progression [2].

Polymerases poly (ADP-ribose) PARP is a family of seventeen proteins that react with poly or mono ADP-ribosylation, and are involved in numerous cellular processes such as DNA repair, cell death, transcription, translation, cell proliferation, or cell response to oxidative stress. PARPs have the ability to modulate the transcriptional functions of both tumor suppressors and oncogenes which affects the ability of PARP to elicit contextual proton and antineoplastic effects [3].

PARP1, PARP2, and PARP3 are involved in the repair of DNA damage. Hence, it is recommended to use PARP inhibitors (PARPi) in the treatment of tumors, in particular those that do not have the ability to recombine (homologous recombination - HR) due to the mutations causing loss of BRCA1 or BRCA2 function. This phenomenon is referred to as synthetic lethality [4–6].

In many types of tumors, elevated *PARP1* mRNA is associated with poor prognosis and thus a shorter survival time. Increased expression of *PARP1* has been demonstrated in various types of tumors, including breast cancer, soft tissue sarcomas, endometrial adenocarcinoma, gliomas, colorectal cancer, myelodysplastic syndrome, neuroma, malignant lymphoma, testicular cancer, ovarian cancer [7–11]. *PARP1* was also found in acute myeloid leukemia and is suggested to be an independent prognostic factor in AML [9, 10].

Hyperactivation of PARP pathway observed in tumors can be used to selectively kill tumor cells. PARPi treatment was shown to be effective in monotherapy and in combination therapies mainly in gynecological cancers, and researchers also suspect potential of PARP inhibitors involvement in acute myeloid leukemia [9, 12, 13].

Recent studies show that PARP may also be involved in the epigenetic regulation maintaining stem cell pluripotency, and their expression is probably necessary for the proper differentiation of stem cells, including hematopoietic stem cells. Some authors even suggest that PARP can be used to reprogram somatic stem cells towards induced pluripotent stem cell (iPSC) [14–18].

Transient receptor potential (TRP) channels are cation channels associated with cancer. To date, TRPM members including TRPM2, 4, 5, 7 and 8 have also been proven to be associated with the proliferation and survival of cells. Among TRP channels, TRPM2 is expressed in many noncancerous cells, such as the brain and peripheral blood cells. Studies show that TRPM2 is associated with various types of cancers, too [19].

TRPM2 is a member of the TRP protein superfamily of ion channels that can be activated by ADP-ribose, β-NAD, TNF-α, and H_2_O_2_ which results in increased values of intracellular free calcium concentration ([Ca ^2+^]_i_) [20]. TRPM2 is a non-selective cationic, Ca^2 + _^ permeable pore, and contains a unique C-terminal region exhibiting ADP-ribose (ADPR) hydrolase activity. The highest expression levels of TRPM2 are observed in the cells of neuronal origin and in the myeloid lineage [21].

Data suggest that TRPM2 channels function as ‘death channels’, because as a matter of fact, heterologous expression of TRPM2 in human embryonic kidney cells or A172 human glioblastoma cells facilitates oxidative stress-induced cell death. Furthermore, expression of TRPM2 has been demonstrated in several tumors such as hepatocellular carcinoma, prostate cancer, lymphoma, leukemia, and lung cancer cell lines in which TRPM2 reportedly may foster cell death [22]. TRPM2 is one ion channel that can be altered to increase apoptosis in cancer cells [23].

The aim of the study was to evaluate of *PARP1, PARP2, PARP3* and *TRPM2* gene expression at mRNA level in the cells of the hematopoietic system of the bone marrow in patients with acute myeloid leukemia, bone marrow from healthy patients, peripheral blood of healthy individuals, and hematopoietic stem cells from the peripheral blood after mobilization.

## Methods

The study comprised a group of 84 patients. The study group consisted of 53 patients hospitalized at the Department and Clinic of Hematooncology with Bone Marrow Transplantation, Medical University of Lublin, Poland, among them 14 patients with acute myeloid leukemia - samples of bone marrow were examined, and 39 patients in remission stage who had previously suffered from hematologic illnesses - samples of hematopoietic stem cells from the peripheral blood which underwent mobilization were tested, 10 patients of the Chair and Clinic of Traumatic Surgery and Emergency Medicine, Medical University of Lublin. The patients had normal bone marrow sampled during hip replacement surgery, and 21 healthy volunteers from whom peripheral blood was collected. The study groups were similar in terms of age and gender.

The material from patients with acute myeloid leukemia was collected at the time of diagnosis, before the treatment was initiated. The AML group consisted of 7 women and 7 men, aged 19–68. The patients’ mean survival was 27 months and 16 days, the shortest survival time was 14 days, the longest 84 months, and over 5-year- survival was observed in 5 out of 14 patients examined. Patient characteristics are included in the Table 1.

**Table 1:** Characteristics of patients with acute myeloid leukemia

Karyotype abnormalities	Therapy	Cause of death
+ 8	DAC	Sepsis after induction therapy
Normal	DAC, consolidation, AlloHSCT	GvHD, infection complications
Unknown	DAC	Sepsis after induction therapy
Unknown	CLAG	Refractoriness on therapy
Inv(16)	DAC, consolidation	Alive
Complex changes	CLAG	Refractoriness on therapy
Normal	DAC, consolidation	Relapse
t(8;21)	DAC, consolidation	Alive
-Y	DAC, consolidation	Aspergillosis
Complex changes	DAC, CLAG-M, consolidation, AlloHSCT	Relapse
Normal	DAC, consolidation, AlloHSCT	Relapse
Complex changes	DAC, consolidation, AlloHSCT	Relapse
Normal	DAC, CLAG-M	Refractoriness on therapy, aspergillosis
Normal	DAC, Consolidation, AlloHSCT	Relapse

The research was approved by the Local Bioethics Commission, Medical University of Lublin (decision No KE-0254/110/2012). The examinations were carried out with the consent of the Clinic managers, all participants signed informed consent to collect the material and conduct the examinations. The research material was collected in the years 2012–2014.

### Cell isolation

Mononuclear cells were isolated from the bone marrow and peripheral blood collected on EDTA anticoagulant by density gradient centrifugation using Histopaque-1077 reagent (Sigma, USA), and PBS reagent (Biomed, Poland).

Hematopoietic stem cells were isolated from the peripheral blood on the fifth day after mobilization with granulocyte growth factor using a cell separator method. The procedure of stem cell separation was performed in the Department of Haematooncology and Bone Marrow Transplantation, Medical University of Lublin [24].

### RNA isolation

RNA was isolated from the samples obtained from blood and marrow mononuclears. Isolation of the total cellular mRNA was carried out by the modified method of Chomczyński and Sacchi [25] using TRI-Reagent (Sigma, USA), isopropanol (Sigma, USA), and chloroform (Sigma, USA). The concentration and purity of the isolated RNA was tested by spectrophotometry (NanoDrop 2000c).

### Reverse transcription reaction

A specific fragment of the analyzed gene was obtained by amplification using cDNA synthesized on the basis 1 μg of isolated RNA using commercially available Reverse Transcriptase Kits (Applied Biosystem, USA) according to the manufacturer’s recommendations. The reverse transcription reaction was carried out in a volume of 20 μl, consisting of: 2 μl (10xRT Buffer), 0.8 μl (10xdNTPs (100 mM)), 2 μl (10xRT Random Primer), 1 μl (RNasin 40 U / μl), 1 μl (Reverse transcriptase 50 U / μl), 3.2 μl (RNAz- and DNAz-free ultrapure water), and 10 μl (1 μg RNA dissolved in 10 μl ultrapure water).

The reaction was carried out in a thermal cycler (Verit Thermal Cycler, Life Technologies), initially the reaction mixture was incubated for 10 min at 25 °C, then for 2 h at 37 °C, followed by 5 min at 95 °C. The resulting cDNA was used for real-time PCR.

### qPCR reaction

The analysis of gene expression was performed by real-time PCR on StepOnePlus (Applied Biosystems) using commercially available TaqMan probes (Applied Biosystems, USA) for endogenous control gene: *GAPDH*: Hs99999905_m1; for *TRPM2* gene: Hs01066071_m1; for *PARP1* gene: Hs00242302_m1 for *PARP2* gene: Hs00193931_m1 for *PARP3* test gene: Hs00193946_m1.

The qPCR reaction was carried out in 96-well-optical plates, in the volume 0f 25 μl / well, consisting of 10.25 μl RNAz- and DNAz-free ultrapure water, 1.25 μl gene-specific probe, 12.5 μl TaqMan Gene Expression Master Mix (Applied Biosystems, USA), and 1 μl cDNA synthesized in the reverse transcription reaction. The qPCR reaction, after the initial 10-min denaturation at 95 °C, was carried out according to the following scheme—40 cycles: 15 s at 95 °C, and 60 s at 60 °C.

To determine the initial amount of cDNA for a given gene in the tested samples, CT (cycle threshold) values were determined, i.e. the number of the PCR cycle after which the fluorescence exceeded the threshold line value. The value of the threshold line (treshold) was determined in the logarithmic growth phase for all tested samples after crossing the base line - the background line, determined separately for each of the samples. To normalize the expression of test genes the CT value against glyceraldehyde 3-phosphate dehydrogenase (GAPDH) reference gene was determined for each sample. Final gene expression was determined against the sample used to calibrate the entire experiment (calibrator - control sample of the peripheral blood from healthy people without leukemia).

The level of relative gene expression was calculated from the formula RQ = 2^-ΔΔCt^ [26]. Data analysis was done by Expression Suite Software 1.0.3. (Life Technologies). Detailed procedures for the cells isolation, reverse transcription and real time PCR reactions have been described in our previous work, where we showed that the studied hematopoietic stem cells of peripheral blood and healthy bone marrow cells express high level of *BIRC5* gene expression, which confirms native nature of the tested hematopoietic stem cells [27].

### Statistical analysis

Statistical analysis was done by Statistica 13 software. To examine the differences between the study groups, ANOVA Kruskal-Wallis Test and U Mann- Whitney Test were applied and the Spearman’s correlation test, *p* < 0.05 was assumed statistically significant.

## Results

**Fig. 1:**
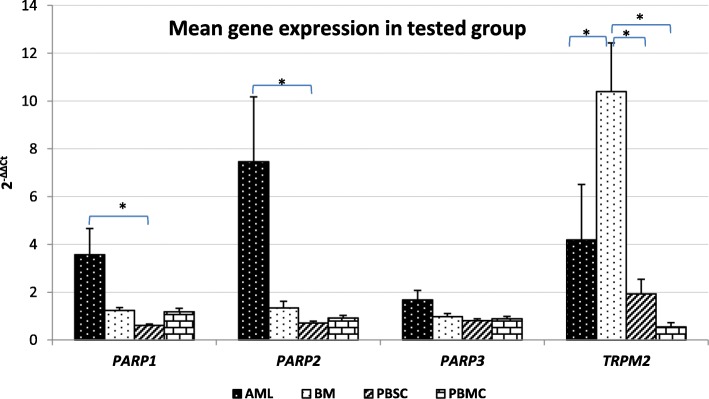
Mean *PARP1, PARP2, PARP3* and *TRPM2* gene expression (2^-ΔΔCt^ ± SE) in groups of cells from the peripheral blood of healthy patients (PBMC), bone marrow of healthy patients (BM), bone marrow cells of acute myeloid leukemia (AML) patients and the peripheral blood stem cells after mobilization (PBSC), *p < 0.05

The results found *PARP1, PARP2, PARP3,* and *TRPM2* genes were expressed in all examined cells. The highest *PARP1* gene expression was found in acute myeloid leukemia cells (AML), while the lowest *PARP1* gene expression was detected in the group of hematopoietic stem cells collected from the peripheral blood after mobilization (PBSC). A statistically significant difference in the expression of *PARP1* between AML group and PBSC was demonstrated. The analysis of *PARP2* gene expression produced similar results, the highest expression was detected in AML group, while PBSC group showed the lowest expression, the difference was statistically significant. Statistical analysis of *PARP3* gene expression in the studied groups did not show significant differences. *TRPM2* gene expression was significantly different in the study groups. The highest expression was recorded in the bone marrow cells (BM), while the lowest in the peripheral blood cells (PBMC), and acute myeloid leukemia. There were significant differences in *TRPM2* gene expression between BM and AML groups, BM and PBSC, and BM and PBMC groups (Fig. 1, Table 2).

**Table 2:** Mean *PARP1, PARP2, PARP3,* and *TRPM2* gene expression (2^-ΔΔCt^ ± SE) in tested groups

Mean gene expression in tested group								
Gene	Group	N	Mean 2^**-ddCT**^	SE	***p***-value			
					AML	PBMC	PBSC	BM
***PARP1***	AML	14	3.572	1.099		0.156	**0.001**^**a**^	0.455
	PBMC	21	1.185	0.146	0.156		0.968	0.899
	PBSC	39	0.615	0.054	**0.001**^**a**^	0.968		0.379
	BM	10	1.243	0.121	0.455	0.899	0.379	
***PARP2***	AML	14	7.463	2.709		0.163	**0.003**^**a**^	0.224
	PBMC	21	0.923	0.110	0.163		0.992	0.988
	PBSC	39	0.709	0.086	**0.003**^**a**^	0.992		0.799
	BM	10	1.349	0.271	0.224	0.988	0.799	
***PARP3***	AML	14	1.684	0.394		0.858	0.700	0.973
	PBMC	21	0.888	0.101	0.858		1.000	0.989
	PBSC	39	0.808	0.083	0.700	1.000		0.990
	BM	10	0.984	0.126	0.973	0.989	0.990	
***TRPM2***	AML	14	4.191	2.317		0.997	0.841	**0.016**^**a**^
	PBMC	21	0.549	0.178	0.997		0.828	**0.025**^**a**^
	PBSC	39	1.934	0.612	0.841	0.828		**0.033**^**a**^
	BM	10	10.392	2.039	**0.016**^**a**^	**0.025**^**a**^	**0.033**^**a**^	

*ANOVA Kruskal-Wallis Test

**Fig. 2:**
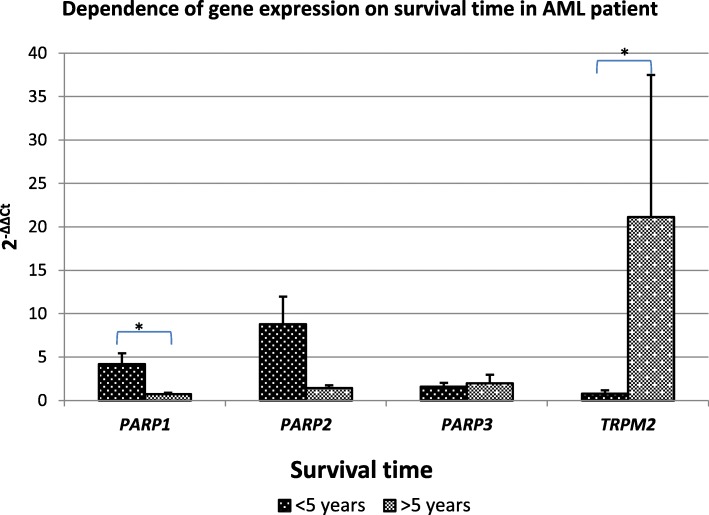
Mean *PARP1*, *PARP2*, *PARP,* and *TRPM2* gene expression (2^-ΔΔCt^ ± SE) in cell groups of bone marrow of acute myeloid leukemia (AML) patients depending on survival time (> 5 years, < 5 years) *p < 0.05

The analysis of the dependence of gene expression and survival of AML patients showed statistically significantly higher *TRPM2* expression and lower *PARP1* expression in the bone marrow cells in patients whose overall survival was longer than 5 years, compared with patients whose overall survival was shorter than 5 years (Fig. 2, Table 3.A.). In the case of *PARP2* and *PARP3* expression, no significant correlation was found with the survival of AML patients. In addition, a statistically significant strong positive correlation of survival with *TRPM2* gene expression and a statistically significant strong correlation of survival with *PARP1* gene expression was demonstrated (Table 3.B.).

**Table 3:** **A.** Mean *PARP1, PARP2, PARP3,* and *TRPM2* gene expression (2^-ΔΔCt^ ± SE) in groups depending on survival time (< 5 years, > 5 years), * U Mann Withney Test. **B.** Correlation of *PARP1, PARP2, PARP3* and *TRPM2* gene expression with survival time *p < 0.05, Spearman’s correlation test

A. Mean gene expression in tested group depending on survival time					B. Correlations between gene expresssion and survival time
Gene	Survival time [years]	Mean 2^**-ΔΔCt**^	SE	***p***-value	survival time
***PARP1***	< 5	4.201	1.254	0.049*	−0.732*
	> 5	0.737	0.161		
***PARP2***	< 5	8.799	3.160	0.422	−0.033
	> 5	1.453	0.312		
***PARP3***	< 5	1.594	0.446	0.559	0.141
	> 5	1.983	1.002		
***TRPM2***	< 5	0.803	0.393	0.013*	0.633*
	> 5	21.134	16.354		

Statistically significant correlations between the expression of the examined genes were demonstrated. Very strong positive correlations were found between the expression of all tested genes: *PARP1* and *PARP2, PARP1* and *PARP3*, *PARP1* and *TRPM2*, *PARP2* and *PARP3* and *TRPM2, PARP3* and *TRPM2* in the group of stem cells collected from the peripheral blood after mobilization Also in the group of bone marrow-derived stem cells, statistically significant positive correlations were observed between the expression of all tested genes: *PARP1* and *PARP2, PARP1* and *PARP3, PARP1* and *TRPM2*, *PARP2* and *PARP3, PARP2* and *TRPM2, PARP3* and *TRPM2*. In the group of hematopoietic stem cells derived from the peripheral blood after mobilization there was a statistically significant positive correlation between *PARP1* and *PARP2* gene expression, *PARP1* and *PARP3*, and *PARP3* and *PARP2, PARP3* and *TRPM2.* In acute myelogenous leukemia group, there were no statistically significant relationships between the expression of the examined genes (Table 4).

**Table 4:** Correlation of *PARP1, PARP2, PARP3,* and *TRPM2* gene expression in tested group, *p < 0.05, Spearman’s correlation test

Correlation of ***PARP1, PARP2, PARP3*** and ***TRPM2*** gene expression in tested group					
Gene	Group	*RQ PARP1*	*RQ PARP2*	*RQ PARP3*	*RQ TRPM2*
*RQ PARP1*	AML		0.489	0.541	−0.564
*RQ PARP2*		0.489		0.301	−0.075
*RQ PARP3*		0.541	0.301		−0.201
*RQ TRPM2*		−0.564	−0.075	− 0.201	
*RQ PARP1*	PBMC		0.965*	0.950*	0.890*
*RQ PARP2*		0.965*		0.991*	0.911*
*RQ PARP3*		0.950*	0.991*		0.951*
*RQ TRPM2*		0.890*	0.911*	0.951*	
*RQ PARP1*	PBSC		0.778*	0.693*	0.284
*RQ PARP2*		0.778*		0.820*	0.236
*RQ PARP3*		0.693*	0.820*		0.398*
*RQ TRPM2*		0.284	0.236	0.398*	
*RQ PARP1*	BM		0.929*	0.964*	0.607*
*RQ PARP2*		0.929*		0.821*	0.571*
*RQ PARP3*		0.964*	0.821*		0.536*
*RQ TRPM2*		0.607*	0.571*	0.536*	

## Discussion

Observed by us positive correlations between *PARP1, PARP2, PARP3,* and *TRPM2* genes expression in the group of mature mononuclear cells derived from the peripheral blood and in the group of bone marrow-derived stem cells suggest that in physiological state there is mutual positive regulation of *PARPs* and *TRPM2* genes expression. In the bone marrow cells of the patients with acute myelogenous leukemia, significant correlations were not observed between the expression of the examined genes. Our research suggests that there is a different regulation of *PARP1, PARP2, PARP3,* and *TRPM2* genes expression in acute myelogenous leukemia cells.

Interestingly, the bone marrow cells of patients with acute myelogenous leukemia show over expression of *PARP1* and *PARP2* genes and decreased *TRPM2* gene expression.

In the hematopoietic stem cells derived from the normal marrow and peripheral blood after mobilization, the opposite situation was observed, i.e. *TRPM2* gene showed increased expression while *PARP1* and *PARP2* gene expression was reduced. Our studies may suggest disrupted signaling between *TRPM2* and *PARP* genes in cancer cells. In addition, the determined correlation between reduced *TRPM2* gene expression and decreased *PARP1* gene expression and shorter overall survival time indicates the prognostic significance of the expression of these genes in AML.

Similarly, the study by Zeng et al. [20] showed that knocking down *TRPM2* increased PARP activity in prostate cancer cells through a unresolved mechanism [20]. In recent years, scientists have devoted much attention to the role of TRP channels in cancer. Among other things, it has been shown that TRPM2 is involved in cell migration and cell death, which are the key processes of cancer cell death [28]. The results of the studies by Zeng et al. [20] showed that in non-cancerous cells, TRPM2 proteins are mainly located at the plasma membrane where they mediate sodium and calcium influx on oxidative stimulation. Cation influxes may lead to membrane depolarization and changes in calcium homeostasis that can lead to programed cell death. In cancer cells, the role of TRPM2 as a plasma membrane ion channel is less important because of the internalization and nuclear localization of TRPM2. Numerous TRPM2 proteins are translocated into the nucleus where they may have an important enzymatic function related to cancer cell proliferation [20].

The cited studies also confirm our analyses in which we showed that in tumor cells *TRPM2* gene expression is significantly decreased whereas *PARP1* and *PARP2* expression is significantly increased, which may indicate that *TRPM2* gene loses its function in tumor cells. In addition, we have shown that in the physiological state there is a positive relationship between *PARP1, PARP2,* and *TRPM2*, which is disturbed in cancer cells.

Correct regulation between the examined genes is extremely important in the context of cell functioning, and its dysregulation may block apoptotic cell death, affect the progression of the cell cycle and increase cell proliferation as well as increase the possibility of DNA repair by overexpressing PARP. On the one hand, the activity of polymerase poli(ADP-ribose) depends on the intracellular calcium concentration, on the other hand polymers of (ADP-ribose) synthesized by PARP1, PARP2 and PARP3 stimulate TRPM2 in the normal cells [20].

The researchers observed that the expression of *TRPM2* in AML subgroups is the higher the more differentiated AML cells are [21]. Klumpp et al. demonstrated that TRPM2 plays a key role in response to DNA damage in leukemic T lymphocytes. The researchers argue that irradiated Jurkat cells use TRPM2 channels to control cell cycle arrest in the G2/M phase [22]. Sumoza-Toledo et al. have demonstrated that the low *TRPM2* expression may be used to predict adverse prognosis in ER-HER + breast cancer. According to the researchers, TRPM2 is a promising biomarker of aggressiveness for breast cancer, and a potential target for new therapies [29]. TRPM2 channel has been identified as playing an important role in several types of cancers [30] including breast cancer, neuroblastoma, prostate cancer, head and neck cancer, melanoma [23], and colorectal cancer [31–33]. Potential TRPM2 involvement was confirmed in cell proliferation, facilitation of cell survival, prostate cancer, melanoma, genomic stability of breast cancer cells, promotion, survival and metastases in the head and neck [19]. The researchers observed that after TRPM2 silencing, the level of DNA damage in breast cancer cells was significantly increased, which was not observed in non-cancer breast cells after similar therapy [33]. So we speculate that therapy directed simultaneously at *TRPM2* and *PARPs* would cause the cancer cells death as a result of damage accumulation and the inability to DNA repair.

Many studies indicate that TRPM2 is an ion channel that is modulated, and that can be altered to increase apoptosis in cancer cells including acute leukemia cells [1]. Based on the conducted studies, we noted that the bone marrow and blood cell samples of the patients with acute leukemia showed statistically significantly lower mean *TRPM2* gene expression compared to the normal bone marrow. Reduced expression of *TRPM2* gene may be associated with disordered apoptotic cycle in cancerous cells.

Our results of *TRPM2* gene expression presented the context of survival time of AML patients showed a statistically significant correlation: the higher *TRPM2* gene expression in bone marrow cells at the time of diagnosis, the longer the mean survival time of the patients. A statistically significantly higher level of *TRPM2* gene expression was observed in the patients whose survival was longer than 5 years, compared to the patients whose survival was less than 5 years. The presented results suggest that the expression of *TRPM2* gene in the marrow cells of AML patients is a possible prognostic marker.

Our results show also increased expression of *PARP1* and *PARP2* gene in AML cells. In many studies *PARP1* expression was found to be significantly increased in several malignant tissues, e.g. breast, uterine, lung, ovarian. Within breast infiltrating ductal carcinoma samples tested, mean PARP1 expression was significantly higher compared to normal breast tissue [34].

The latest data also confirm that PARP1 was highly expressed in cytogenetically normal AML patients and AML cell lines compared to normal bone marrow cells. It may indicate that PARP1 plays a critical role in the development of AML [4]. Research by Fonfria et al. [35] confirms the hypothesis that PARP enzyme activity is a central component of the pathway linking oxidative stress with TRPM2 activation [35]. Intracellular Ca^2+^ regulation has a crucial role in tumorigenesis, including cell replication and apoptosis. Nuclear expression of *TRPM2* may also influence nuclear Ca^2+^ concentration [20].

In our studies we showed that in the bone marrow cells of patients with acute myeloid leukemia, *PARP1* and *PARP2* genes are overexpressed and *TRPM2* gene is down-regulated, which may suggest disturbed signaling between these genes. The modified TRPM2 function may suggest disturbed signaling between TRPM2 and PARPs at the transcript level, which increases the expression of polymerases. Numerous studies have reported that overexpression of *PARP* genes in turn is associated with uncontrolled cell proliferation, increased DNA repair ability, and increased telomerase activity which is associated with cell immortalization [36].

The demonstrated in our study positive correlations between *PARP1, PARP2, PARP3,* and *TRPM2* gene expression in the group of PBMC and in the group of BM cells suggest that in the physiological state there is mutual positive regulation of *PARP* family members and *TRPM2* gene expression. In the bone marrow cells from the patients with acute myelogenous leukemia, significant relationships between the expression of the test genes were not observed. Our research suggests that there is a different regulation of *PARP1, PARP2, PARP3*, and *TRPM2* gene expression in acute myelogenous leukemia cells. We speculate that correct signaling between *TRPM2* and *PARPs* is essential for physiology, and its loss may affect the development of acute leukemia. Regulation of *PARP1, PARP2, PARP3,* and *TRPM2* genes expression may provide a new therapeutic strategy against AML.

Moreover, our studies have shown that there is a negative correlation between *PARP1* gene expression and overall survival in the group of cells from the marrow of AML patients. It has been shown that the higher *PARP1* expression at the mRNA level, the shorter the patients’ survival. In the group of patients whose survival was over 5 years, *PARP1* expression was statistically significantly lower in comparison with patients whose overall survival was less than 5 years, which confirms the prognostic significance of *PARP1*.

## Conclusions

Our research suggests that in physiological conditions the cells of the hematopoietic system exhibit mutual positive regulation of *PARP1, PARP2, PARP3,* and *TRPM2* gene expression. In acute myelogenous leukemia cells, *PARP1, PARP2,* and *TRPM2* gene mRNA levels deregulate. Furthermore, survival of AML patients is associated with the expression of *PARP1* and *TRPM2* genes determined at the time of diagnosis. Therefore, there is a need for further research on the mutual regulation of *PARP1, PARP2, PARP3,* and *TRPM2* gene expression in AML.

## Data Availability

The datasets used and/or analysed during the current study are available from the corresponding author on reasonable request.

## Abbreviations

PBMC: Peripheral blood mononuclear cells
BM: Bone marrow
AML: Acute myeloid leukemia
PBSC: Peripheral blood stem cells
DAC: Daunorubicine+cytarabine,+cladribine
CLAG: Cladribine+cytarabine+G-CSF
CLAG-M: CLAG+mitoxantrone
AlloHSCT: Allogeneic stem cell transplantation
GvHD: Graft versus host disease
